# On the Causes of Death in Lobar Pneumonia

**Published:** 1920-06

**Authors:** F. H. Edgeworth

**Affiliations:** Physician to the Bristol Royal Infirmary and Professor of Medicine in the University of Bristol


### ON THE CAUSES OF DEATH IN LOBAR PNEUMONIA.

BY

F. H. Edgeworth, M.A., M.D., D.Sc.,

*Physician to the Bristol Royal Infirmary and Professor of Medicine in the University of Bristol.*

---

During this last winter I have seen several cases of collapse occurring at the end of lobar pneumonia, and have been struck by the absence of any effective treatment. Such cases have long been known. They were described by Fagge in the words, " It now and then happens that the occurrence of the crisis is followed by great prostration and collapse from which the patient never recovers."

It seemed worth while to find out what are the characteristics of the condition and how frequently it occurs. To do so I have analysed all the cases of lobar pneumonia—from the age of 12 years upwards—which came into my wards at the Royal Infirmary during the last ten years. The cases recorded are consecutive, with the exception that all entering between October, 1918, and April, 1919, are excluded owing to the then prevalence of influenza, and to the difficulty or impossibility in some cases of distinguishing between extensive broncho-pneumonia and lobar pneumonia. The cases numbered 135, and the average age of the patients was 29 years. Seventeen died. This gives a mortality of 12.5 per cent.—about 8 per cent. less than is stated, in Osler, to be the rule for hospital patients of this age. The average age of those who died was 40 years, and no patient died under the age of 20. This is in accordance with the general law that the mortality increases with years.

As regards general treatment. Once the diagnosis was certain the patient was disturbed as little as possible, *e.g.* was not raised in bed for daily examination of the back. He was allowed to be in bed with the number of pillows which gave the greatest comfort. If, as often happened, the sputum was so viscid that expectoration was difficult, citrate of sodium, in 20 or 30 grain doses, was given every four hours until it was easy to bring up. It was found that larger doses than this were apt to produce diarrhœa. If this occurred it was checked by chloride or lactate of calcium. Opium was useless. If the pulse rose to 120 per minute a hypodermic injection was given every two hours, alternately of ♏3 of Liq. Strych. hydrochlor. and gr. i. of camphor dissolved in Ol. Olivæ. This appeared to be distinctly useful, though it did not raise the blood-pressure.

Pleural effusion occurred in four cases. In two pus was found—these were operated on and drained. In two cases the fluid withdrawn by an exploring syringe was cloudy, and was aspirated, ℥x. in one case, ℥xx. in the other. All four recovered.

The causes of death were as follows. Of one (in 1916) I have no notes recorded. One died on the thirteenth day from gangrene of the right lower lobe. One died on the twenty-first day, with a persistent consolidation of the left lower lobe, irregular fever, and expectoration of a sputum containing pneumococci only. One died, quite suddenly, on the sixth day, of no apparent cause during life or explanation post-mortem. One died from pericarditis. Seven died, feverish, cyanosed and dyspnœic, at various dates from the seventh day onwards. Six of these showed, post-mortem, hepatisation of either both lower lobes, or of the whole of both lungs. They had been treated by the administration of oxygen five minutes every quarter of an hour. Possibly, but I do not feel sure, the persistent administration of oxygen by Haldane's apparatus might—if we had had it then—have saved these patients.

In one case, aged 50, with consolidation of the right lower lobe, the temperature was persistently low, about 101°, and death occurred on the seventh day. He showed no response to the infection.

This leaves four cases—about 3 per cent.—in which death occurred apparently as the result of a persistent fall in the blood-pressure. In three this happened during the fever, in one just when the temperature had fallen by crisis to the normal. The cases, then, described by Fagge do not form, on the average, more than 1 per cent. of the total.

In general the systolic blood-pressure in cases of lobar pneumonia is about 90 mm. Hg. In these cases, just described, it falls during the course of a few hours to 60 mm., and then death occurs. There does not appear to be any detectable difference between the phenomena in those patients in whom the temperature is still high—103° or over—and those in whom it has just fallen to normal. In both the cause of death appears directly attributable to the fall in the blood-pressure, so that the circulation is no longer carried on. The cause of this fall is doubtful—whether due to a failure of blood-pressure-raising hormones such as those of the pituitary and suprarenal bodies, or to a general poisoning of the muscle-fibres of the cardio-vascular apparatus. There is, as far as I know, no direct evidence on the matter.

The practical question is how to counteract this dangerous fall in blood-pressure. Strychnine, digitalis, and camphor appear to be useless. My experience with adrenalin hydrochloride has been unfortunate. In two cases (treated subsequently to the end of last year and so not in the above statistics) ♏5 of 1 in 1000 strength every quarter of an hour failed to raise the blood-pressure. Possibly the dose was insufficient. In any case, however, physiological experiments show that the continual administration of adrenalin hydrochloride will not keep the blood-pressure high. Tyramine acts better. In one case of pneumonia I found that gr. $\frac{1}{3}$ injected hypodermically raised the blood-pressure from 80 to 105 mm. Hg. within two hours, and its administration every four hours kept the blood-pressure up. On the other hand, in two cases, seen for the first time at the end of the crisis, with a fallen blood-pressure—as estimated by the finger—tyramine failed to prevent death. The drug thus seems useful if used directly the blood-pressure begins to fall. If this has already fallen to a dangerous point, no treatment I know of will prevent a fatal termination.

---

